# Extraventricular Neurocytoma With a Complex Presentation: The Role of Molecular Studies and Technology in Its Management

**DOI:** 10.7759/cureus.86229

**Published:** 2025-06-17

**Authors:** Jose Valerio, Jorge Zumaeta, Guillermo de Jesus Aguirre Vera, Pablo Mazon, Noe Santiago Rea, Andres M Alvarez-Pinzon

**Affiliations:** 1 Department of Neurosurgery, Neurosurgery Oncology Center of Excellence, Miami Neuroscience Center at Larkin, South Miami, USA; 2 Department of Neurosurgery, Neurosurgery Oncology Center of Excellence, Miami Neuroscience Center at Larkin, South Miami, PER; 3 Vascular, Tumor and Functional Neurosurgery, Guillermo Almenara Irigoyen National Hospital, Lima, PER; 4 Neurosurgical Oncology, Latinoamerica Valerio Foundation, Miami, USA; 5 School of Medicine, School of Medicine and Health Sciences of Tecnológico de Monterrey "Ignacio A. Santos", Mexico City, MEX; 6 Neurological Surgery, Latinoamerica Valerio Foundation, Weston, USA; 7 Neurological Surgery, Hospital Insular de Gran Canaria, Las Palmas, ESP; 8 Neurosurgery Oncology, Latinoamerica Valerio Foundation, Weston, USA; 9 Cancer Neuroscience, The Institute of Neuroscience of Castilla y León (INCYL) University of Salamanca (USAL), Salamanca, ESP; 10 Neurosurgery Oncology, Baptist MD Anderson Cancer Center, Jacksonville, USA

**Keywords:** central nervous system tumor, diagnostic modalities, gamma knife (gk) radiosurgery, intraventricular neoplasm, navigation surgery, neurocytoma, specific molecular diagnosis

## Abstract

Diagnosis and management of central nervous system tumors is challenging, especially when they have unusual presentation and require molecular analysis to confirm the pathology to offer the best treatment with the least risk of morbidity and mortality. This is a 34-year-old man who presented with a hemorrhagic lesion at the corpus callosum and intraventricular level that was initially classified as an ependymoma but was later redefined as a neurocytoma after molecular analysis. The patient had no significant medical history. The initial clinical picture was manifested with intense headache, nausea, and photophobia. Initial management consisted of placement of an external ventriculostomy for acute hydrocephalus. A biopsy of the lesion, aided by navigation, was performed. A ventriculoperitoneal shunt for persistent hydrocephalus was placed. After confirming the molecular diagnosis of neurocytoma, treatment with a gamma knife provided excellent results. The importance of this case centers around the fundamental role of molecular diagnosis in accurately classifying central nervous system (CNS) tumors, specifically in cases where histopathological analysis may be inconclusive, allowing for better directed management. Technology enables effective management of these complex lesions through minimally invasive treatment options like the gamma knife.

## Introduction

Extraventricular neurocytomas (EVNs) are rare neuronal tumors that pose significant diagnostic challenges due to their histopathological similarities with other central nervous system (CNS) neoplasms. Initially classified in the 2007 World Health Organization (WHO) classification of CNS tumors, these tumors typically present as large, well-circumscribed lesions in the cerebral hemispheres, with a predilection for the frontal and parietal lobes. However, they have been reported in various locations, including the thalamus, cerebellum, and even the spinal cord, highlighting their diverse anatomical distribution and clinical presentations [1].

Imaging plays a pivotal role in the initial evaluation of EVNs. Magnetic resonance imaging (MRI) typically reveals heterogeneous lesions with variable contrast enhancement, often accompanied by cystic components and calcifications [2-5].

Histopathologically, EVNs exhibit a wide spectrum of morphologies, including sheets, clusters, ribbons, or rosettes of monotonous neurocytes interspersed with neuropil [1,6]. This variability can lead to misdiagnosis, as these characteristics are often like other kinds of CNS tumors, such as ependymomas.

The advent of molecular profiling, particularly DNA methylation analysis, has revolutionized the diagnosis and classification of CNS tumors by distinguishing them from histologically similar entities [1,5]. The detection of the FGFR1: TACC1 fusion provided crucial evidence for the diagnosis of EVNs. Recent research has underscored the importance of such molecular alterations in not only diagnosis but also in understanding the pathogenesis and potential therapeutic targets for these rare tumors [2,7].

This case highlights the critical interplay between imaging, histopathology, and molecular profiling in diagnosing EVNs, emphasizing the necessity of a multidisciplinary approach involving neuroradiology, neuropathology, and molecular genetics. While gross total resection remains the cornerstone of treatment, the infiltrative nature and proximity of these tumors to vital structures often necessitate adjuvant therapies such as radiation or emerging targeted treatments based on molecular alterations. Despite advancements in classification and management, gaps remain in understanding the biology and optimal therapeutic strategies for EVNs. By presenting a detailed analysis of this case, including diagnostic challenges and molecular insights, this report aims to contribute to the growing literature on EVNs and guide future research efforts to improve patient outcomes.

## Case presentation

A 34-year-old male from Honduras with no relevant clinical history. His clinical presentation began with an intense headache associated with nausea, vomiting, and photophobia. He was initially given a brain MRI in his country of origin, which showed a mass at the level of the corpus callosum with intraventricular hemorrhage and acute hydrocephalus. A right frontal external ventriculostomy was placed to temporarily resolve the hydrocephalus. The patient was then transferred to the USA for specialized management.

**Figure 1:**
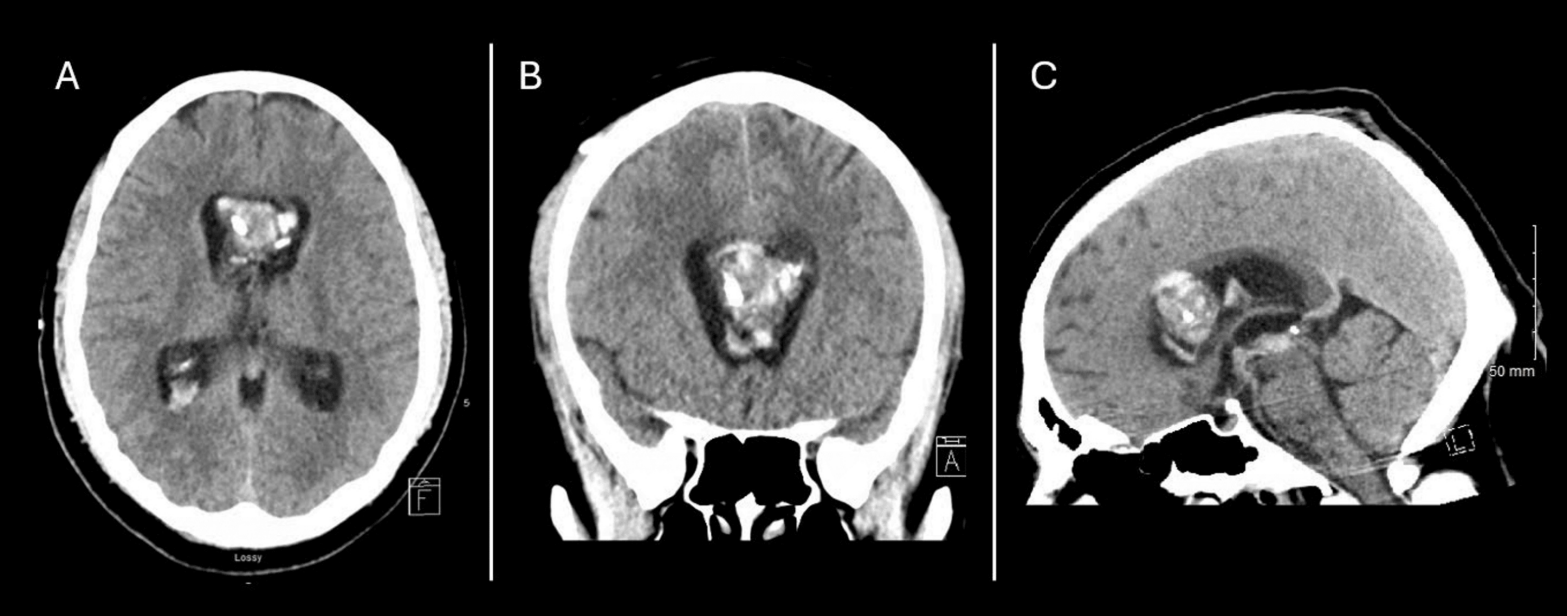
Initial CT Imaging Demonstrating Intraventricular Hemorrhage with Obstructive Hydrocephalus Initial brain CT scan shows intraventricular hemorrhage in the lateral ventricles with associated ventriculomegaly. An external ventricular catheter is also observed in the right frontal horn, in direct contact with the tumor mass (A and B). Obstruction at the level of the cerebral aqueduct due to intraventricular hemorrhage is seen (C).

Upon arrival at our hospital, the patient was awake, alert, and oriented. On physical examination, he did not present motor deficits, and the external ventriculostomy was functioning. Imaging tests of brain tomography and MRI were performed. The head CT showed a mass at the level of the corpus callosum that extended to the intraventricular space. He showed signs of hemorrhage and punctate calcifications. The external ventriculostomy was in the right frontal horn of the lateral ventricle, in direct contact with the tumor mass (Figure 1).

**Figure 2:**
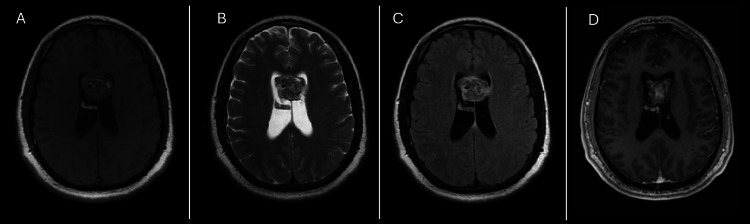
MRI Characteristics of Midline Intraventricular Lesion Involving the Septum Pellucidum and Corpus Callosum Brain MRI showing a lesion of 2.5 × 3.0 × 2.1 cm located in the midline anterior body and frontal horns of the lateral ventricles straddling the septum pellucidum and extending into the splenium of the corpus callosum with heterogeneous hyperintense signal on the T1 imaging (A), mixed signal intensity on T2 sequence as in FLAIR (B, C), and bubbly heterogenous enhancing lesion in the T1 with contrast sequence (D).

Brain MRI showed a heterogeneous lesion between the anterior third and middle third of the corpus callosum with intraventricular extension. A slight contrast enhancement was seen in the central part of the lesion (Figure 2). The patient was scheduled for stereotactic biopsy of the brain lesion with the aid of navigation.

Histopathological and immunohistochemical analysis

**Figure 3:**
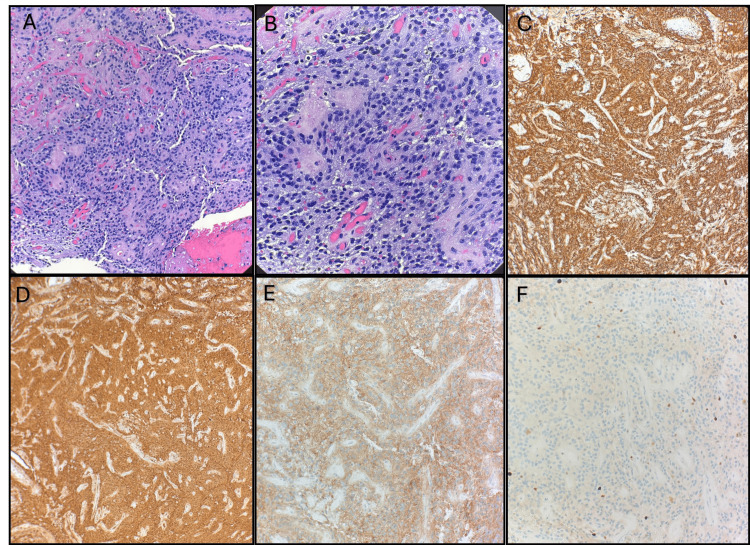
Histopathological and Immunohistochemical Features of a Neuroepithelial Neoplasm with Glial Differentiation Pathologic Anatomy: A and B) Neuroepithelial neoplasm with morphologic appearance of ependymoma formed by mildly to moderately pleomorphic tumor cells in a fibrillary glial matrix with vascular pseudo-rosettes (H&E 100× and 400×). C) GFAP (glial fibrillary acidic protein) immunohistochemical stain strongly highlights the cells of the neuroepithelial neoplasm. D) S-100 immunohistochemical stain strongly highlights the cells of the neuroepithelial neoplasm. E) Synaptophysin immunohistochemical stain strongly highlights the cells of the neuroepithelial neoplasm. F) KI67 proliferation index is low, highlighting 4-6% of the neoplastic cells.

A stereotactic biopsy of the lesion was performed, which initially suggested an ependymoma (CNS WHO Grade 2). The tumor demonstrated a mild to moderate pleomorphism, a fibrillar glial matrix, and characteristic perivascular rosettes. Immunohistochemical analysis supported the classification with strong diffuse positivity for glial fibrillary acidic protein (GFAP) and S100, as well as synaptophysin expression. This suggested the possibility of neuronal differentiation. The Ki-67 proliferation index was approximately 5%, which indicated a low-grade neoplasia (Figure 3).

**Figure 4:**
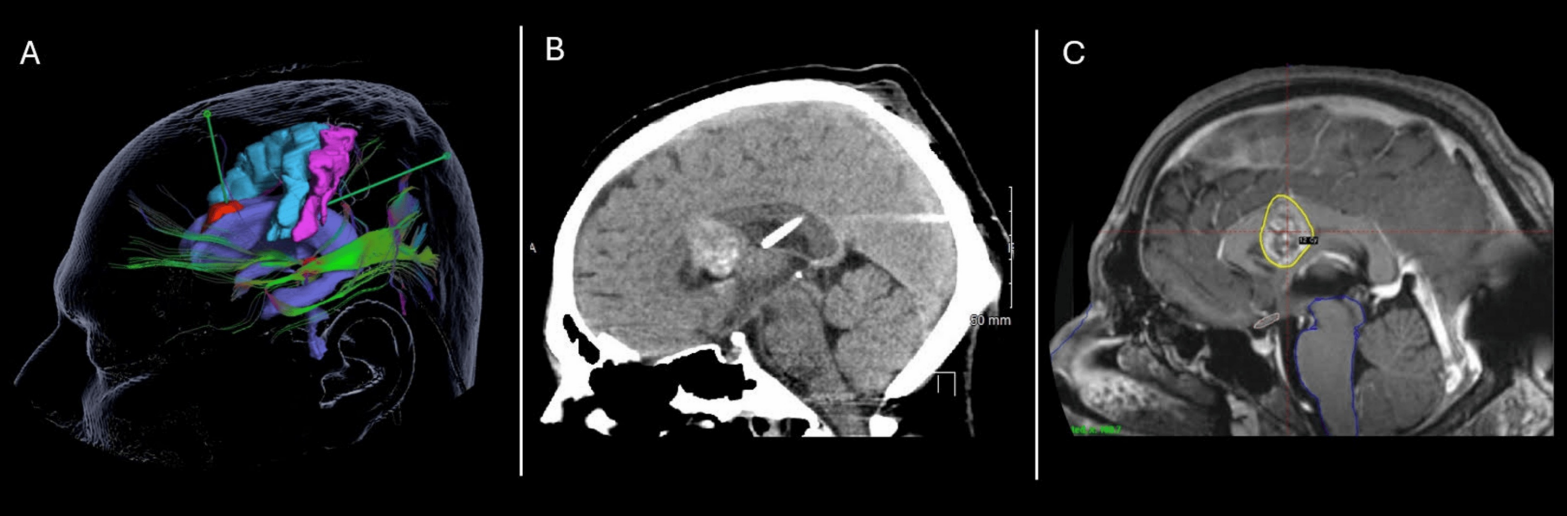
Surgical Planning for the Brain Biopsy Trajectory, Ventriculoperitoneal Shunt Placement, and Gamma Knife Radiosurgery. (A) The image shows the two trajectories created for the biopsy and ventriculoperitoneal shunt placement, taking into account the motor and sensory cortices, as well as the optic radiation. (B) Shows a sagittal view with the intraventricular placement of the shunt. (C) Radiosurgery planning which includes the entire lesion as the target.

Due to the persistence of hydrocephalus, a definitive ventriculoperitoneal shunt was required, which was also planned using navigation. (Figure 4A). The surgery was uneventful, and the patient was transferred to the intensive care unit in stable condition. 

The patient evolved favorably after the surgical procedures, achieving improvement of the headache and neurological stability. Valproic acid was used for seizure prophylaxis, and steroids for the treatment of cerebral edema. Follow-up imaging studies showed a decrease in ventricular dilation (Figure 4B). The patient was discharged from the hospital for outpatient follow-up, pending the results of the biopsy.

**Figure 5:**
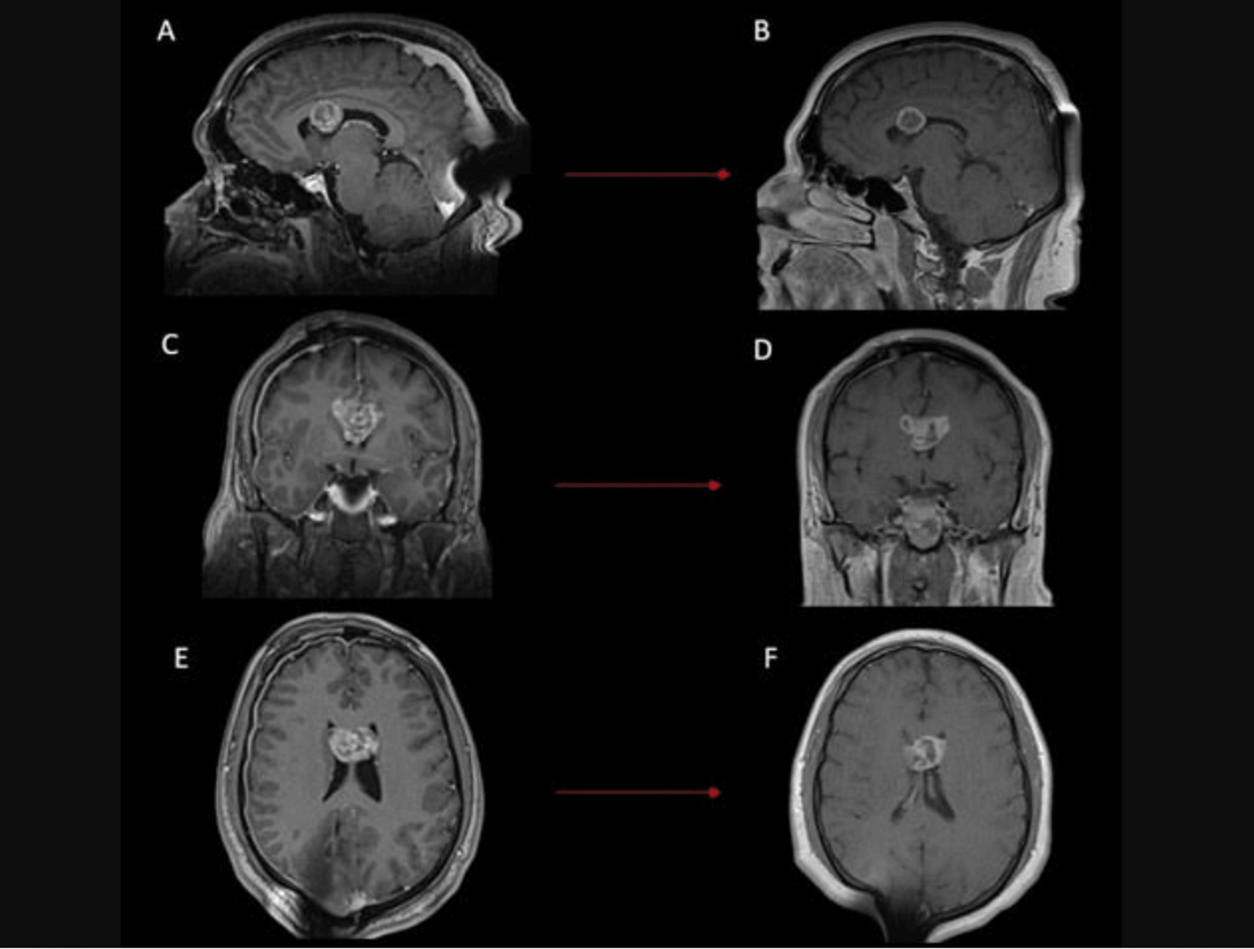
MRI Comparison Before and After Gamma Knife Treatment Showing Lesion Volume Reduction Brain MRI comparing pre-treatment imaging with the 3-month follow-up. (A) and (B) show the sagittal view, (C) and (D) the coronal view, and (E) and (F) the axial view. The images demonstrate a reduction in lesion volume along with central necrosis.

The diagnostic workup for this patient involved a combination of neuroimaging, histopathologic analysis, and molecular studies to accurately determine the nature of the tumor. After obtaining the definitive diagnosis of EVN, the patient was scheduled for Gamma Knife Radiosurgery (GKRS) with a good clinical outcome (Figure 4C). The patient was seen in the clinic asymptomatic without any neurological deficit, with follow-up MRI showing a significant decrease in tumor volume without any associated brain or spinal cord lesions (Figure 5).

Diagnostic evaluation and treatment

Initial Imaging and Clinical Findings

The presentation of acute headaches and signs of hydrocephalus motivated the development of urgent neuroimaging studies. The CT scan of the brain revealed an intraventricular hemorrhage in the lateral and third ventricles, which was accompanied by ventriculomegaly. The magnetic resonance characterized a mass-like lesion with enhancement in the anterior body of the lateral ventricles, with perilesional edema and associated mass effect.

Molecular and Methylation Profile

Given the overlapping histopathological features and the presence of neuronal markers, additional molecular studies were performed. The methylation profile of the DNA reclassified the tumor as an extraventricular neurocytoma, CNS WHO Grade 2. This classification was reinforced by the detection of FGFR1: TACC1 fusion, which is a molecular marker which is characteristic of neurocytomas. These molecular findings helped to explain the unusual histopathological presentation, which initially suggested an ependymoma because of its similar morphology.

Final diagnosis

The integration of the histological, immunohistochemical, and molecular data allowed for the establishment of the diagnosis of extraventricular neurocytoma, CNS WHO Grade 2. This case underscores the critical role of advanced molecular diagnostics in the accurate classification of CNS tumors. With a deep insight into the importance of advanced molecular diagnostic techniques to aid in the classification of challenging CNS tumors. This is especially important for cases with overlapping histopathological characteristics.

Initially, the histopathological study favored a diagnosis of grade II ependymoma based on morphology and immunohistochemistry. Nonetheless, studies of methylation revealed a molecular classification of extraventricular neurocytoma, CNS WHO grade 2. This process underlines the necessity for a multidisciplinary approach. This combines neuropathology, molecular genetics, and neuroradiology to ensure accurate diagnosis and optimal patient management.

Gamma Knife Radiosurgery

The treatment plan consisted of a single site with 16 isocenters utilizing the 4 mm, 8 mm, and 16 mm collimators. The patient was then transferred to the gamma knife suite, where radiosurgical treatment was administered. A total dose of 12 Gy was delivered to the 50% isodose line, encompassing a treatment volume of 8.2 cc (Figure 3C). The maximum dose to the brainstem was less than 1.6 Gy, remaining well within safe tolerance limits.

Upon completion of the radiosurgical procedure, the patient was taken to the procedure room for removal of the stereotactic head frame. The pin sites were thoroughly cleansed and appropriately dressed. Following a short period of observation and monitoring, the patient was discharged home in stable condition with a prescription for dexamethasone.

## Discussion

The 2021 WHO Classification of CNS Tumors classifies neurocytomas into two main groups, central neurocytomas (CN) and EVNs, both considered rare intracranial pathologies. The prevalence of CN and EVNs is notably low, accounting for approximately 0.1% to 0.5% of all brain tumors. The combined annual incidence rate for CN and EVNs in the United States is reported at 0.032 per 100,000 population, with CN having a higher incidence (0.022) compared to EVN (0.009) [8].

Neurocytomas are diagnosed through a combination of clinical evaluation strategies. MRI is the primary imaging modality utilized in the identification of these lesions. Regarding CN, its intraventricular location is characteristic, commonly within the lateral ventricles. Its presentation is a well-defined enhancing lesion, with occasional signs of calcifications and cystic components [2,3]. On the other hand, EVNs typically arise outside the ventricular system, most frequently found in the frontal lobe and cerebellum, with some cases reported in the temporal lobe. They often exhibit heterogeneous contrast enhancement, cystic changes, perilesional edema, and calcification on MRI [9]. Histopathological examination has common characteristics, including round cells with clear cytoplasm and round nuclei, which are arranged in rosettes. Positive neural markers like synaptophysin, NeuN, and neuron-specific enolase (NSE), with variable Ki-67 indices of proliferation, are also characteristic [10]. Molecular analysis is centered on DNA methylation and next-generation sequencing, which serves to help in diagnosis confirmation, particularly due to the similarities in histopathological features from other tumors. This gives diagnostic clarity and helps determine the probable prognosis [11,12].

CN associate symptoms related to high intracranial pressure, including headache, nausea, and vomiting [13]; these symptoms commonly mask obstructive hydrocephalus, which is provoked by an impediment of the cerebrospinal fluid flow caused by the location of the tumor in the lateral ventricles [14-16]. On the other hand, EVNs’ clinical presentation includes a variety of symptoms such as headaches, nausea, and neurological deficits according to its localization, mass, and edema effect [17]. Specific case reports have described intracerebral hemorrhage as the initial presentation of EVNs [18], like this case presentation.

Given that our case involved a complex hemorrhagic lesion located at the level of the corpus callosum, with involvement of the ventricular space, the use of advanced technologies such as navigation was essential for accurate biopsy and precise placement of the ventriculoperitoneal shunt. This approach, supported by current technology, minimizes risks and ensures favorable postoperative outcomes without causing additional urological complications [19].

Ependymoma is diagnosed through a combination of clinical evaluation, histopathology, and molecular testing. MRI is the primary modality utilized to identify the location and the extent of the tumor. The characteristics of the ependymoma are a heterogeneous solid-cystic appearance with calcifications and multiple enhancement patterns [20,21]. Histopathological analysis is crucial for a proper diagnosis. Ependymomas may be identified and classified in terms of cellular morphology and mitotic activity. It common histopathological features include perivascular pseudo-rosettes and ependymal rosettes, with immunohistochemical markers such as GFAP and epithelial membrane antigen (EMA) being frequently positive [22]. It is important to note that histopathology alone is not regarded as a sufficiently accurate tool to confirm the diagnosis [23,24]. Molecular testing is becoming a valuable tool for the diagnosis of challenging tumors in the CNS. WHO classification divides ependymomas into different subgroups, which are based upon molecular markers and specific anatomical localizations. Supratentorial ependymomas are classified through ZFTA, previously known as RELA, or YAP1 fusions. While the posterior Fossa (PF) ependymomas are denominated into PFA and PFB depending on methylation profiles, spinal ependymomas may show MYCN amplification [25]. DNA methylation profiling has become a progressively more valuable tool for accurate diagnosis and prognosis. This helps to distinguish ependymomas from other tumors which has other similar histopathological characteristics, while also helping to disclose an analysis for potential treatment strategies [26,27].

As in this case presentation, there are specific challenges to correctly diagnose this kind of lesion given its close similarity in clinical presentation, as in MRI characteristics and histological features between ependymoma and neurocytoma. Diffusion-weighted imaging in combination with an apparent diffusion coefficient (ADC) value could help to aid differentiation. Ependymomas are commonly of a more elevated ADC values when compared to neurocytomas, which show smaller ADC values provoked by a denser cellularity [28]. Both types of tumors exhibit small, round cells with clear cytoplasm, making the differentiation an arduous process without certainty on the diagnosis. The use of immunohistochemical staining is necessary. Particularly, neurocytomas show a high positivity for neural markers such as synaptophysin and NeuN. On the other hand, ependymomas could show different glial markers, specifically GFAP. It is important to note that the markers are not fully specific and may overlap [6,29]. Regarding molecular studies, the FGFR1: TACC1 fusion in neurocytomas is an essential molecular alteration due to its impact on the clinical implications. This leads to the production of chimeric proteins that have oncogenic potential. It is associated with an uncontrolled activation of the FGFR1 kinase domain, which is a promoter of oncogenesis through signaling of survival and proliferation [30-32]. The presence of these mutations can lead to the proper diagnosis of neurocytomas. The presence of this mutation alters the prognosis due to its implications on the risk of recurrence and the enhanced aggressiveness; proper understanding of molecular characteristics is key to personalizing the follow-up strategy properly [30,31]. This mutation also possesses implications for the potential treatment strategy. Since FGFR inhibitors like erdafitinib and pemigatinib have shown high effectiveness in the treatment of FGFR alterations. The identification of FGFR1: TACC1 fusions in neurocytomas could provide, in the future, a targeted treatment strategy which may be more effective than traditional therapeutic options [32,33].

There are specific challenges to be considered regarding molecular classification. The 2021 WHO Classification of Tumors of the CNS places an emphasis on the growing relevance of molecular diagnostic tools to enhance diagnostic accuracy. The ependymomas are classified on the basis of specific gene alterations, such as ZFTA or YAP1 fusions being characteristic in supratentorial ependymomas, and MYCN amplification being the norm in spinal ependymomas. The neurocytomas could necessitate methylation profiling to be able to distinguish them from tumors that have histological features with similarities [23,24,34]. The use of an integrative diagnostic approach, which comprises histopathological, immunohistochemical, and molecular data, is progressively becoming more relevant for certainty in the diagnosis. This approach aids in overcoming the limitations of the diagnostic methods alone, providing a comprehensive understanding of the nature of the tumor [6,24].

The proper differentiation of diagnoses is important due to the differences in the prognosis and treatment strategies for ependymoma versus extraventricular neurocytoma. Regarding the supratentorial ependymomas with confirmed ZFTA fusion regularly pose a worse prognosis when they are compared to those with YAP1 fusion [23].

The primary treatment of ependymomas is through maximal safe resection, which is then followed by radiotherapy. This is especially true for grade II and III tumors or in cases in which complete resection is not feasible or not achieved. Chemotherapy is commonly used in recurrences, in which emerging immunotherapies are currently being investigated for specific molecular subtypes [35,36].

Regarding neurocytoma, these tumor types commonly have a better prognosis compared to ependymomas, but still have challenging management. The survival (OS) rate for EVN is approximately 90.4% over 5 years. But the progression-free survival is 48.6%, which indicates a substantial risk of recurrence. Patients younger than 50 years have better outcomes [37].

Although considered benign tumors, neurocytomas have various biological behaviors, histological patterns, and clinical courses. In the last 15 years, fractionated radiotherapy and radiosurgery, in addition to microsurgery, have been used in their management [38].

The rarity of EVN has thus far hindered a precise definition of its surgical characteristics and treatment strategy. They have a worse prognosis than CNs, despite both being WHO grade 2 tumors. Most series address the treatment of CNs, so management guidelines are extrapolated to EVN. Patients with CN who underwent subtotal resection (STR) followed by either conventional radiotherapy or stereotactic radiosurgery achieved outcomes comparable to those undergoing gross total resection (GTR). However, the outcomes of tumors with EVN remain uncertain [39].

GKRS has shown favorable results in CN as a primary or adjuvant treatment. However, data on EVN are lacking due to its extremely low incidence [40]. Favorable results of GKRS treatment have been reported for residual tumors after CN surgery [41]. Favorable results for GKRS have also been reported in radiologically diagnosed CN, suggesting that GKRS can be used as a primary treatment for small CNs [42].

A recent systematic review on the management of EVNs reports that complete surgical removal is the cornerstone of treatment and that there is no clearly established role for adjuvant postoperative therapy, but each case should be treated individually [37].

Treatment consisting of GTR offers a significant improvement in survival outcomes compared with subtotal resection. However, when the tumor is located near eloquent areas, surgery may carry significant risks, including postoperative neurological deficits [38]. Stereotactic radiosurgery is especially advantageous for lesions in eloquent locations due to its ability to minimize damage to surrounding brain structures, offering an effective alternative as a primary or adjunctive treatment with a lower risk of neurological complications [40,41]. Balancing tumor control with preservation of neurological function remains a major challenge in the comprehensive treatment of neurocytomas [40,42].

Systematic analyses of clinical outcomes following stereotactic radiosurgery (SRS) for CN have suggested that this approach may be an effective therapy. Current reported doses are considered safe and effective, respectively. Radiation-associated adverse events, defined as hyperintensity around the treated lesion on imaging, are rare after SRS for central neurocytoma [43]. A 20% local control failure rate has been reported for patients treated with primary SRS versus a 40% failure rate in patients treated with adjuvant SRS [44]. Other publications have also found primary SRS to be effective in controlling asymptomatic incidental NC [45]. Smaller tumor volumes are less likely to require surgical decompression or be symptomatic, as a smaller tumor volume significantly correlates with better local control [43].

GKRS as primary treatment for CN has been reported to achieve successful long-term disease control. In a study of 14 patients treated primarily with GKRS, none experienced recurrence during a median follow-up of 25 months. Furthermore, no patient developed distant recurrences or spread along the craniospinal axis after GKRS [41].

The choice between surgery and radiosurgery depends on several factors, including the size and exact location of the tumor and the patient’s general condition. For this reason, each case must be managed individually [37]. In our case, when presenting a bleeding lesion at the level of the corpus callosum that led to persistent hydrocephalus, we had to resort to the use of navigation technology to accurately take a biopsy of the lesion and then place a ventriculoperitoneal shunt in the right occipital horn of the lateral ventricle to avoid contact with the tumor mass. The ability to place a catheter with high precision through a posterior approach in cases of hemorrhagic lesions located in the anterior ventricular region highlights the critical value of image-guided technology. As demonstrated in our case, a posterior approach would have been the most appropriate initial strategy to minimize the risk of contact with the tumor mass that occurred during the initial surgical intervention. The location and extension of the lesion required a minimally invasive treatment with less possibility of complications, so having the final diagnosis of EVN, we proceeded to perform the gamma knife treatment, obtaining excellent results in disease control and a favorable neurological evolution. Based on the data reviewed and the case presented, we can confirm that stereotactic radiosurgery represents an effective and safe therapeutic option for the treatment of EVNs in eloquent areas, offering significant advantages in terms of precision and functional preservation, as in the case reported here.

## Conclusions

This case underscores the importance of a multidisciplinary approach in diagnosing and treating complex CNS tumors, such as neurocytomas, particularly when histopathological findings overlap with other entities and the clinical presentation is atypical. Initially diagnosed as a grade 2 ependymoma, molecular methylation analysis reclassified the tumor as a neurocytoma. This distinction carries significant clinical implications, as prognosis and treatment strategies differ between these entities. The identification of the FGFR1: TACC1 fusion and methylation profiling was pivotal in achieving an accurate diagnosis, highlighting the critical role of molecular evaluation as a complement to histopathology in CNS tumor diagnostics. The occurrence of an EVN in the corpus callosum with intratumoral hemorrhage is a rare and complex presentation. The use of advanced technologies, such as neuronavigation and GKRS, enhances patient safety by facilitating biopsy procedures, hydrocephalus management, and precise treatment delivery with minimal morbidity and mortality.
